# Predictors of survival after surgery with curative intent for perihilar cholangiocarcinoma

**DOI:** 10.1186/s12957-020-02060-x

**Published:** 2020-11-03

**Authors:** Joachim Geers, Joris Jaekers, Halit Topal, Raymond Aerts, Cindy Vandoren, Guy Vanden Boer, Baki Topal

**Affiliations:** 1 Department of Visceral Surgery, University Hospitals KU Leuven, Herestraat 49, 3000 Leuven, Belgium; 2 Management Information & Registration, University Hospitals KU Leuven, Herestraat 49, 3000 Leuven, Belgium

**Keywords:** Biliary tract cancer, Liver resection, Perihilar cholangiocarcinoma, Survival, Prognostic factors

## Abstract

**Background:**

Several clinicopathological predictors of survival after curative surgery for perihilar cholangiocarcinoma (pCCA) have been identified; however, conflicting reports remain. The aim was to analyse clinical and oncological outcomes after curative resection of pCCA and to determine prognostic factors.

**Methods:**

Eighty-eight consecutive patients with pCCA underwent surgery with curative intent between 1998 and 2017. Survival curves were estimated using the Kaplan-Meier method and compared using the log-rank test. Twenty-one prognostic factors were evaluated using multivariate Cox regression models.

**Results:**

Postoperative complications were observed in 73 (83%) patients of which 41 (47%) were severe complications (therapy-oriented severity grading system (TOSGS) grade > 2), including a 90-day mortality of 9% (*n* = 8). Overall survival (OS) and disease-free survival (DFS) rates at 5 and 10 years after surgery were 33% and 19%, and 37% and 30%, respectively. Independent predictors of OS were locoregional lymph node metastasis (LNM) (risk ratio (RR) 2.12, confidence interval (CI) 1.19–3.81, *p* = 0.011), patient American Society of Anesthesiologists (ASA) physical status classification system > 2 (RR 2.10, CI 1.03–4.26, *p* = 0.043), and depth of tumour penetration (pT) > 2 (RR 2.58, CI 1.03–6.30, *p* = 0.043). The presence of locoregional LNM (RR 2.95, CI 1.51–5.90, *p* = 0.002) and caudate lobe resection (RR 2.19, CI 1.01–5.14, *p* = 0.048) were found as independent predictors of DFS.

**Conclusions:**

Curative surgery for pCCA carries high risks with poor long-term survival. Locoregional LNM was the only predictor for both OS and DFS.

## Background

Perihilar cholangiocarcinoma (pCCA) or Klatskin tumour is a rare malignancy and is defined as an adenocarcinoma that occurs in the biliary tract at the confluence of the hepatic ducts [1]. It has an annual incidence of about 1–2 in 100,000 in Western countries and accounts for up to 50% of all CCAs [1–3]. Radical surgical resection is the only curative option but has a high postoperative morbidity and mortality rate. The 5-year overall survival (OS) ranges from 13 to 42% for resectable pCCA [4], whereas the median OS of patients with unresectable pCCA is about 12 months with no survivors at 5 years [5]. The poor survival rates after potentially curative surgery are due to cancer recurrence as a result of the aggressive biological behaviour of pCCA with early hematogenous, lymphatic, and perineural spread of cancer cells [6–8]. Postoperative mortality and morbidity rates vary from 5 to 18% and from 60 to 70%, respectively, which reflect the complexity and technical challenge of the surgical management of pCCA [5].

Although several clinicopathological factors have been associated with long-term survival after potentially curative surgery for pCCA, there are conflicting reports regarding these prognostic factors. A recent meta-analysis tried to identify prognostic factors that may determine overall survival after surgery for pCCA, underlining the need for studies to find a homogeneous group of prognostic factors that could be used after potentially curative treatment of patients with pCCA [9]. The aim of the current study was to analyse clinical and oncological outcome of patients with pCCA after surgery with curative intent and to determine prognostic factors for both overall (OS) and disease-free (DFS) survival.

## Methods

### Patients

Between January 1998 and December 2017, a total of 88 consecutive patients underwent a surgical resection with curative intent for histopathologically proven pCCA and were included for further analysis. Data were collected in a prospective database and studied retrospectively. Patients with intrahepatic cholangiocarcinoma, distal cholangiocarcinoma, or gallbladder carcinoma were excluded. The resection margin status was examined and sorted into R0- (microscopic tumour-free margins), R1- (microscopic residual tumour in the margin), and R2-resection status (macroscopic residual tumour in the margin). Patients who underwent a palliative resection (R2-resection) were also excluded from the study population. Patient characteristics are shown in Table 1.

**Table 1:** Patient and tumour characteristics

Variables	***n =*** 88 (range, %)	Missing data, ***n*** (%)
Age (years, median, range)	64 (30–80)	0 (0)
Gender		0 (0)
Male	52 (59)	
Female	36 (40)	
BMI (kg/m^2^, median, range)	24.54 (17.6–35.4)	8 (9)
ASA		0 (0)
1	6 (7)	
2	53 (60)	
3	29 (33)	
Cholangitis	12 (14)	0 (0)
Biliary drainage	56 (64)	0 (0)
Endoscopic	44 (50)	
Percutaneous transhepatic	2 (2)	
Both	10 (11)	
CEA serum level (μg/L)	2.55 (0.7–18.8)	50 (57)
CA19-9 serum level (kU/L)	151 (1–3215)	36 (41)
Bismuth-Corlette classification		0 (0)
Type I	12 (14)	
Type II	19 (22)	
Type IIIa	20 (23)	
Type IIIb	25 (28)	
Type IV	12 (14)	
Pathological T-stage		0 (0)
Tis	1 (1)	
T1	12 (14)	
T2	65 (74)	
T3	6 (7)	
T4	4 (5)	
Pathological N-stage		4 (5)
pN0	46 (52)	
pN1	33 (38)	
pN2	5 (6)	
Differentiation grade		24 (27)
Well (G1)	20 (23)	
Moderate (G2)	30 (34)	
Poor (G3)	14 (16)	
Tumour diameter (mm, median, range)	26 (10–80)	19 (22)
Total number of lymph nodes (median, range)	8 (1–22)	7 (8)
Positive lymph nodes (median, range)	2 (1–13)	1 (1)
ECLNI	14 (16)	1 (1)
Perineural invasion	69 (78)	2 (2)
Resection margin		0 (0)
R0	74 (84)	
R1	14 (16)	
pRM		38 (43)
≤ 1 mm	36	
> 1 mm	14	

*BMI* body mass index, *ASA* American Society of Anesthesiologists physical status classification system, *CEA* carcinoembryonic antigen, *CA* carbohydrate antigen, *Tis* tumour in situ, *ECLNI* extracapsular lymph node involvement, *pRM* magnitude of tumour-free resection margin

### Peri-operative management

Serum levels of carcinoembryonic antigen (CEA) and carbohydrate antigen 19-9 (CA19-9) were measured at the time of clinical presentation. Preoperative diagnostic imaging consisted of contrast-enhanced computed tomography (CT) scan of the chest and abdomen, and a cholangiogram either by magnetic resonance imaging (MRCP) or by endoscopic retrograde cholangiopancreatography (ERCP). The tumour was classified according to the modified Bismuth-Corlette classification [10].

Biliary drainage before surgery was performed in patients presenting with cholangitis and/or in patients who were expected to need a major hepatectomy and who had a serum total bilirubin level above 5 mg/dL at the time of presentation. Tumour response to neo-adjuvant systemic chemotherapy was preoperatively evaluated according to the revised RECIST criteria [11]. Postoperative adjuvant systemic chemotherapy was offered to patients who either had an R1-resection or had locoregional lymph node metastasis (LNM), and were fit enough within 3 months postoperative to tolerate chemotherapy.

### Resectability

The main factors that defined resectability were future liver remnant volume and its vascular integrity, and biliary drainage. Major hepatic resection was performed if the preoperative total bilirubin serum level was below 5 mg/dL. A resection of 3 or more Couinaud segments was considered as major hepatectomy [12].

The decision to define patients as no candidates for resection was made in a multidisciplinary setting and based on Jarnagin’s criteria, i.e. metastatic disease, patient factors (medically unfit for surgery, hepatic cirrhosis), and tumour factors (extension to secondary biliary radicles bilaterally, encasement or occlusion of the main portal vein proximal to its bifurcation, atrophy of one hepatic lobe with contralateral portal vein branch encasement or occlusion, atrophy of one hepatic lobe with contralateral tumour extension to secondary biliary radicles, or unilateral tumour extension to secondary biliary radicles with contralateral portal vein encasement or occlusion) [13]. Extra-regional lymph nodes, such as peri-aortic or peri-caval lymph nodes, which were macroscopically suspicious during surgery, were investigated on frozen section pathology. When found positive for tumour involvement, the patient was considered to have metastatic disease and did not qualify for a curative resection. Histopathological findings were reported according to the 8th TNM classification [14].

### Surgical procedure

Staging laparoscopy with intraoperative laparoscopic ultrasound was done routinely to exclude patients with peritoneal or liver metastases. When a curative resection was considered feasible, laparoscopy was converted to a bilateral subcostal laparotomy. The type and extent of resection depended on the modified Bismuth-Corlette stage [10], the macroscopic involvement of the major blood vessels, and the intraoperative frozen section pathological assessment of the biliary surgical margins. Locoregional lymphadenectomy (hilar, pericholedochal, and hepatoduodenal) was performed routinely. Frozen section pathology was done intraoperatively to examine biliary surgical resection margins. A caudate lobe resection and/or vascular resection (portal vein and/or hepatic artery resection) were done only in case of pre- or intraoperative suspicion of tumour involvement. Intra- and postoperative characteristics are shown in Table 2.

**Table 2:** Intraoperative and postoperative characteristics

Variable	***n*** = 88 (range, %)	Missing data, ***n*** (%)
Staging laparoscopy	78 (89)	0 (0)
Type of surgery		0 (0)
Left hepatectomy	29 (33)	
Right hepatectomy	26 (30)	
Central hepatectomy	4 (5)	
Extended left hepatectomy	2 (2)	
Extended right hepatectomy	2 (2)	
Bile duct resection	23 (26)	
Segmental hepatectomy and bile duct resection	2 (2)	
Caudate lobe resection	56 (64)	0 (0)
Common hepatic artery resection	3 (3)	0 (0)
Portal vein resection	18 (20)	0 (0)
Duration of surgery (minutes, median, range)	240 (140–360)	62 (71)
Intraoperative blood loss (mL, median, range)	900 (50–12000)	47 (53)
Intraoperative transfusion	38 (43)	0 (0)
Pringle manoeuvre performed	8 (9)	1 (1)
Total duration (minutes, median, range)	25 (10–45)	2 (2)
Intraoperative complication	20 (23)	0 (0)
Postop ICU admission	16 (18)	0 (0)
Length of ICU stay (days, median, range)	4 (1–13)	0 (0)
TOSGS > 2 (excl. mortality)	33 (38)	0 (0)
Postoperative haemorrhage		0 (0)
ISGLS grade B	5 (6)	
ISGLS grade C	5 (6)	
Liver failure		0 (0)
ISGLS grade A	1 (1)	
ISGLS grade B	2 (2)	
ISGLS grade C	5 (6)	
Bile leak		0 (0)
ISGLS grade A	2 (2)	
ISGLS grade B	18 (21)	
ISGLS grade C	5 (6)	
Postoperative blood transfusion	34 (39)	0 (0)
Postoperative complications		0 (0)
Intra-abdominal abscess	25 (28)	0 (0)
Sepsis	5 (6)	
Portal thrombosis	4 (5)	
Pulmonary complication	12 (14)	
Cardiac complication	4 (4)	
Multiple organ failure	4 (4)	
Reoperation	13 (15)	0 (0)
In-hospital mortality	8 (9)	0 (0)
90-day mortality	8 (9)	0 (0)
Length of hospital stay (days, median, range)	18 (3–94)	0 (0)
Readmission within 30 days of discharge	12 (14)	0 (0)
Any recurrence	42 (48)	
Locoregional recurrence	18 (21)	0 (0)
Location of first metastatic recurrence	39 (44)	0 (0)
Liver	13 (15)	
Peritoneum	12 (14)	
Pulmonary	3 (3)	
Retroperitoneal lymph nodes	6 (7)	
Pancreas	2 (2)	
Abdominal wall muscle	2 (2)	
Colon	1 (1)	

*ICU* intensive care unit, *TOSGS* therapy-oriented severity grading scale, *ISGLS* International Study Group of Liver Surgery

### Statistical analysis

Postoperative complications were classified according to the therapy-oriented severity grading system (TOSGS), with severe complications as grade 3 or higher [15]. Classification systems of the International Study Group of Liver Surgery (ISGLS) were used to classify postoperative haemorrhage, liver failure, and bile leaks accordingly [16–18]. The Kaplan-Meier estimates were used for survival analysis. Overall survival (OS) was defined as time from surgery to date of death. Disease-free survival (DFS) was defined as time from surgery to date of any cancer recurrence or death. Patients were followed up until death or until the date of study closure in December 2018, resulting in a median follow-up time of 23 (0–236) months. Locoregional recurrence was defined as cancer recurrence at the surgical resection site or at the liver hilum. Cancer recurrence more distant in the liver or at other distant sites was defined as metastatic or distant recurrence.

A set of 21 potential prognostic factors were analysed: patient age (years), gender (male/female), body mass index (BMI), American Society of Anesthesiologists (ASA) physical status classification system, the year of diagnosis/surgery (dichotomised in 10- and 5-year groups), presence of cholangitis, preoperative total bilirubin serum level, tumour depth invasion (pT), locoregional LNM (pN+), tumour differentiation grade (pG), tumour diameter (mm), extracapsular lymph node involvement (ECLNI), microvascular invasion, macrovascular invasion, perineural invasion, R-status (R0/R1), magnitude of tumour-free resection margin (pRM), intraoperative transfusion, caudate lobe resection, major hepatectomy, and portal vein resection. Variables missing more than 20% of data were excluded from multivariate analysis. Multivariate analyses were done using the Cox proportional hazards model. A *p* value of < 0.05 was considered statistically significant. Statistical analyses were done using the software package JMP for Mac, version 14 (SAS Institute Inc., Cary, NC, USA).

## Results

### Patients

All patient and tumour characteristics are shown in Table 1. Median age was 64 years (range 30–80), and male-to-female ratio was 1.4:1. Fifty-eight (66%) patients presented with obstructive jaundice, 12 (14%) patients had cholangitis, 11 (13%) patients had a cholestasis-related blood panel, 5 (6%) patients complained of vague abdominal discomfort with weight loss, and 2 (2%) patients were diagnosed during the follow-up of another malignancy. One patient (1%) had an underlying primary sclerosing cholangitis.

### Peri-operative management

The median CEA was 2.6 μg/L (range 0.7–18.8, reference value < 3.8 μg/L), and the median CA19-9 was 151 kU/L (range 1–3215, reference value < 34 kU/L). Forty-four (50%) patients underwent an internal endo-biliary stent via ERCP, 2 (2%) patients had an external drainage via percutaneous transhepatic biliary approach, and 10 (11%) patients were managed with a combination of ERCP and percutaneous approach. Preoperative portal vein embolisation was performed in one patient (1%).

Cisplatin-based systemic chemotherapy prior to surgery was given in 3 patients (3%), resulting in either partial response (*n* = 1) or stable disease (*n* = 2), evaluated according to the revised RECIST criteria [11].

The types of surgical resections are listed in Table 2. The caudate lobe was resected in 56 (64%) patients. Resection of the main portal vein or the contralateral portal vein (contralateral to the hepatectomy) was done in 18 (20%) patients. Histopathological R0-resection was obtained in 74 (84%) patients*.* All procedures were performed by one of the two hepatobiliary surgeons.

Adjuvant gemcitabine or gemcitabine plus cisplatin was given to 13 (15%) patients. Within this subgroup, 10 patients had locoregional LNM and 3 patients had an R1-resection. One of the latter patients also received adjuvant radiotherapy. The majority of patients (*n* = 36) who met the criteria to receive adjuvant therapy (R1-resection *n =* 5, locoregional LNM *n* = 25, both R1-resection and locoregional LNM *n* = 6) were excluded because they were considered not fit enough to tolerate chemotherapy within the first 3 months postoperative.

### Postoperative outcome

Postoperative complications were observed in 73 (83%) patients, including 8 (9%) in-hospital deaths and 33 (38%) severe complications (TOSGS > 2). The 90-day mortality was the same as the in-hospital mortality (*n* = 8, 9%). Causes of postoperative mortality were liver failure (*n* = 3), cardiac arrest (*n* = 2), haemorrhage (*n* = 1), pulmonary embolism (*n* = 1), and undetermined (*n* = 1). Indications for reoperation were bleeding (*n* = 5), biliary fistula (*n* = 4), abscess (*n* = 3), and thoracic empyema (*n =* 1). Postoperative characteristics are shown in Table 2.

### Survival and prognostic factors

Median OS was 30.9 months (range 22.8–44.2). The 1-, 3-, 5-, and 10-year OS rates were 76%, 47%, 33%, and 19%, respectively. Median DFS time was 28.7 months (range 19.5–52.2). The 1-, 3-, 5-, and 10-year DFS rates were 82%, 45%, 37%, and 30%, respectively.

**Fig. 1:**
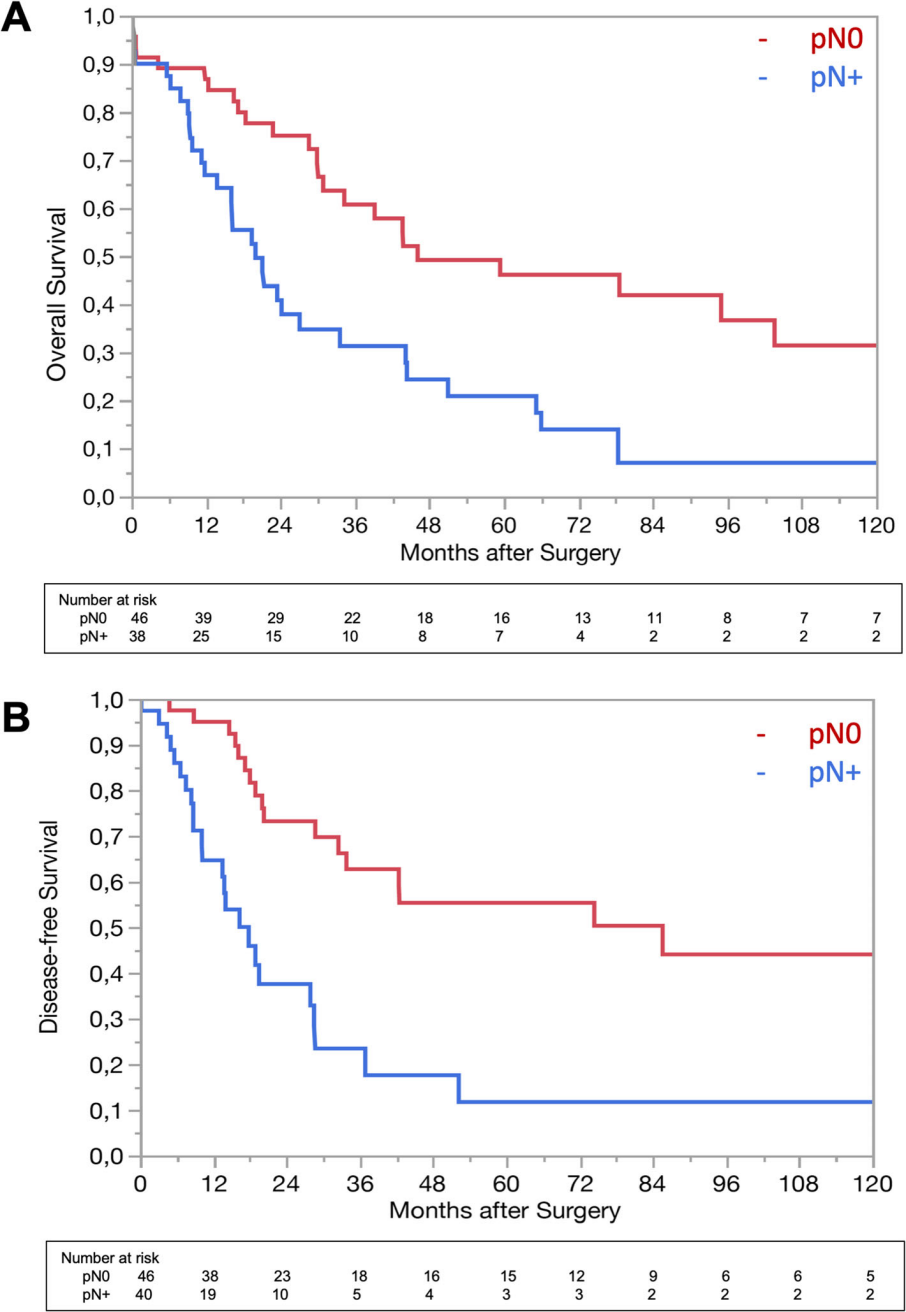
The Kaplan-Meier estimates for overall survival and disease-free survival

In univariable analyses, several potential prognostic factors were significantly related to OS (Table 3) or DFS (Table 4). In multivariable analyses, the presence of locoregional LNM (RR 2.12, CI 1.19–3.81, *p* = 0.011), patient ASA score > 2 (RR 2.10, CI 1.03–4.26, *p* = 0.043), and pT-stage > 2 (RR 2.58, CI 1.03–6.30, *p* = 0.011) were found as independent predictors of poor OS. pN+ (RR 2.95, CI 1.51–5.90, *p* = 0.002) and caudate lobe resection (RR 2.19, CI 1.01–5.14, *p* = 0.048) were found as independent predictors of DFS. The Kaplan-Meier estimates for OS and DFS for both pN0 and pN+ subgroups are shown in Fig. 1. The 5-year OS for patients with pN0 was 46% as compared to 14% for patients with pN+ (*p* < 0.002). The 5-year DFS for patients with pN0 was 55%, compared to 12% for pN+ patients (*p* < 0.0001). In 35% of patients with pN+, cancer recurrence was observed within a year after surgery. In patients with pN0, less than 5% had a recurrence within a year.

**Table 3:** Results of univariable and multivariable analyses for overall survival

Parameter		No. of patients	Univariable	Multivariable	
			*p* value	Risk ratio (95% CI)	*p* value
Patient-related					
Age		88	0.764	1.61 (0.36–7.91)	0.539
Gender	Male	52	0.583	1.21 (0.64–2.24)	0.555
	Female	36			
BMI		80	0.910		
ASA	> 2	29	0.017	2.10 (1.03–4.25)	0.043
	≤ 2	59			
Year of diagnosis (10 years)	1998–2007	33	0.533		
	2008–2017	55			
Year of diagnosis (5 years)	1998–2002	14	0.932		
	2003–2007	19			
	2008–2012	30			
	2013–2017	25			
Cholangitis	Yes	12	0.971		
	No	76			
Serum level of total bilirubin at presentation		88	0.098		
Tumour-related					
Pathological T-stage	> pT2	10	0.002	2.58 (1.03–6.30)	0.043
	≤ pT2	78			
Pathological N-stage	pN+	38	< 0.002	2.12 (1.19–3.80)	0.011
	pN0	46			
Differentiation grading	1	20	0.025		
	2	30			
	3	14			
Tumour size (mm)		69	0.660		
ECLNI	Yes	14	0.736		
	No	25			
Microvascular invasion	Yes	59	0.769		
	No	22			
Macrovascular invasion	Yes	20	0.041	1.63 (0.09–8.27)	0.660
	No	68			
Perineural invasion	Yes	69	0.238		
	No	17			
R-status	1	14	0.318		
	0	74			
pRM	≤ 1 mm	36	0.127		
	> 1 mm	14			
Surgery-related					
Intraoperative transfusion	Yes	38	0.006	1.58 (0.89–2.83)	0.121
	No	50			
Caudate lobe resection	Yes	56	0.111		
	No	32			
Major hepatectomy	Yes	63	0.715		
	No	25			
Portal vein resection	Yes	18	0.020	0.78 (0.13–15.17)	0.828
	No	70			

*RR* risk ratio, *CI* confidence interval, *BMI* body mass index, *ASA* American Society of Anesthesiologists physical status classification system, *ECLNI* extracapsular lymph node involvement, *pRM* magnitude of tumour-free resection margin, *AJCC* American Joint Committee on Cancer

**Table 4:** Results of univariable and multivariable analyses for disease-free survival

Parameter		No. of patients	Univariable	Multivariable	
			*p* value	Risk ratio (95% CI)	*p* value
Patient-related					
Age		88	0.096	1.08 (0.22–5.60)	0.926
Gender	Male	52	0.617	1.10 (0.57–2.08)	0.771
	Female	36			
BMI		80	0.390		
ASA	> 2	29	0.864		
	≤ 2	59			
Year of diagnosis (10 years)	1998–2007	33	0.921		
	2008–2017	55			
Year of diagnosis (5 years)	1998–2002	14	0.256		
	2003–2007	19			
	2008–2012	30			
	2013–2017	25			
Cholangitis	Yes	12	0.217		
	No	76			
Serum level of total bilirubin at presentation		88	0.288		
Tumour-related					
Pathological T-stage	≥ pT3	10	0.100	0.88 (0.29–2.41)	0.813
	≤ pT2	78			
Pathological N-stage	pN+	38	< 0.0001	2.95 (1.51–5.90)	0.002
	pN0	46			
Differentiation grading	1	20	0.275		
	2	30			
	3	14			
Tumour size (mm)		69	0.510		
ECLNI	Yes	14	0.558		
	No	25			
Microvascular invasion	Yes	59	0.507		
	No	22			
Macrovascular invasion	Yes	20	0.021	3.11 (0.00–3.37)	0.273
	No	68			
Perineural invasion	Yes	69	0.241		
	No	17			
R-status	1	14	0.455		
	0	74			
pRM	≤ 1 mm	36	0.002		
	> 1 mm	14			
Surgery-related					
Intraoperative transfusion	Yes	38	0.109		
	No	50			
Caudate lobe resection	Yes	56	0.006	2.19 (1.01–5.14)	0.048
	No	32			
Major hepatectomy	Yes	63	0.260		
	No	25			
Portal vein resection	Yes	18	0.002	1.63 (0.50–2.01)	0.148
	No	70			

*RR* risk ratio, *CI* confidence interval, *BMI* body mass index, *ASA* American Society of Anesthesiologists physical status classification system, *ECLNI* extracapsular lymph node involvement, *pRM* magnitude of tumour-free resection margin, *AJCC* American Joint Committee on Cancer

## Discussion

In this retrospective cohort study, surgery with curative intent for pCCA was associated with 5- and 10-year OS rates of 33% and 19%, and with 5- and 10-year DFS rates of 37% and 30%, respectively. These results are in line with current literature [5, 7, 13, 19–29]. The 5-year OS rates of these larger series range from 22 to 44%. Five-year DFS rates range from 12 to 32%; however, the majority of these studies did not report 5-year DFS rates. Moreover, some of these series also included patients with an R2-resection and the use of (neo-)adjuvant treatment is non-standardised. This makes an adequate comparison between these series difficult. The presence of locoregional LNM (pN+), tumour invasion depth (pT), and ASA score were found to be independent predictors of OS. The presence of locoregional LNM and caudate lobe resection had a negative influence on DFS.

The negative impact of locoregional LNM on survival has already been well-established [4, 8, 9, 19, 23, 25, 27, 29–36]. In the current study, the 5-year DFS of patients without LNM was 55%, compared to 12% of patients with LNM. About a third (35%) of patients with LNM developed cancer recurrence within a year after surgery, whereas cancer recurrence occurred in less than 5% of patients without LNM. These observations provide some support for the statement by Groot Koerkamp et al. that patients with LNM have less chance to be cured [34]. However, in the series of Buettner et al., patients with LNM who underwent a resection still seem to have a better survival after resection compared to patients without surgery [36]. The pT-stage of the AJCC/UICC TNM-staging system was already identified as a prognostic factor [4, 8, 9, 20, 23, 29, 32]. In our cohort, pT-stage was a predictor of OS, but had no effect on DFS.

Several authors support a caudate lobe resection in order to achieve R0-resection and improve survival [21, 22, 24, 37, 38]. In the present study, caudate lobe resection was associated with worse DFS. As we only performed caudate lobe resection in case of suspected tumour involvement, this subgroup of patients may represent a more advanced stage of disease. Despite not performing a routine caudate lobe resection, the 84% R0-resection rate in our study seems to be higher than that in the literature, which ranges from 31 to 71% [21, 22, 37, 38]. However, comparison of our R0-resection status with that in the literature would be methodologically wrong as there are many differences in study populations [24].

In the current study, R0-resection status did not affect OS nor DFS, despite being considered in the literature as one of the primary predictors of survival [4, 7, 13, 19–23, 25–27, 30, 32, 34, 35, 37, 39, 40]. These findings might be explained by the rather small number of patients (*n* = 88) and the relatively low rate of R1-resection (16%) in our study. Moreover, as some patients (*n* = 3) with R1-resection received adjuvant chemotherapy, these results might be biassed. In a recent meta-analysis, the resection margin status had a significant effect on OS but high heterogeneity was reported [9]. Comparable to our findings, Lurje et al. reported that R1-resection was not associated with an impaired outcome [29]. The series of Lee et al. also showed no significant difference for both 3- and 5-year overall survival between patients in the R0-resection group and patients in the R1-resection group [33].

The simultaneous resection of the main or contralateral portal vein was not a predictor of survival in our cohort. We only performed a (main or contralateral) portal vein resection in selected cases, when tumour involvement was suspected. In the trend towards a more aggressive and radical surgical approach for pCCA, an obligatory portal vein resection is advocated by Neuhaus et al. [24]. They state that a ‘hilar-en-bloc resection’ (right trisectionectomy and concomitant portal vein resection) is oncologically superior for pCCA and reported a 5-year survival of 58% in the hilar-en-bloc resection group. However, they only included patients who had an R0-resection for this survival analysis. A portal vein resection is considered to be safe and feasible, but some authors report that it is associated with an increased complication and mortality rate [41, 42].

Regarding the clinical outcomes, there was a major morbidity rate (TOSGS > 2) of 38% and mortality rate of 9%. These are comparable to both Eastern and Western high-volume centres with morbidity rates between 30 and 68% and mortality rates ranging from 2 to 13% [5, 7, 13, 19–29]. The largest contributing factor to the morbidity in our cohort was biliary leakage (ISGLS grade B) and intra-abdominal abscesses requiring drainage. Most of the patients in our study population who died, did so due to liver failure (*n* = 3), which is consistent with the literature [2].

Strengths of this study include its homogeneous study population and long-term follow-up. Most previous reports include patients with intrahepatic or distal CCA, or even gallbladder cancer as well, despite the fact these pathologies are considered to have different behaviour and biology. The current study is however limited by its retrospective design and restricted sample size. Considering the low incidence of pCCA, acquiring a large single-centre cohort is difficult. Despite the homogenous study population, there is still heterogeneity present regarding the surgical procedures. Moreover, because of the long study period, there was no standardised use of (neo-)adjuvant treatment for pCCA, making the interpretation of results difficult.

## Conclusions

Curative surgery for perihilar cholangiocarcinoma remains a high-risk procedure with poor long-term survival. Locoregional LNM was the only significant prognostic factor to determine both OS and DFS.

## Data Availability

The datasets used and/or analysed during the current study are available from the corresponding author on reasonable request.

## Abbreviations

AJCC: American Joint Committee on Cancer
ASA: American Society of Anesthesiologists
BMI: Body mass index
CA19-9: Carbohydrate antigen 19-9
CEA: Carcinoembryonic antigen
CI: Confidence interval
DFS: Disease-free survival
ECLNI: Extracapsular lymph node involvement
ERCP: Endoscopic retrograde cholangiopancreatography
ICU: Intensive care unit
ISGLS: International Study Group of Liver Surgery
LNM: Lymph node metastasis
MRCP: Magnetic resonance cholangiopancreatography
OS: Overall survival
pCCA: Perihilar cholangiocarcinoma
pRM: Pathological resection margin
RECIST: Response evaluation criteria in solid tumours
RR: Risk ratio
Tis: Tumour in situ
TOSGS: Therapy-oriented severity grading scale
UICC: Union for International Cancer Control
